# Immunological Evaluation of Lipopeptide Group A Streptococcus (GAS) Vaccine: Structure-Activity Relationship

**DOI:** 10.1371/journal.pone.0030146

**Published:** 2012-01-12

**Authors:** Mehfuz Zaman, Abu-Baker M. Abdel-Aal, Yoshio Fujita, Karen S. M. Phillipps, Michael R. Batzloff, Michael F. Good, Istvan Toth

**Affiliations:** 1 The University of Queensland, School of Chemistry and Molecular Biosciences (SCMB), St. Lucia, Queensland, Australia; 2 Institute for Glycomics, Griffith University, Gold Coast, Queensland, Australia; Dana-Farber Cancer Institute, United States of America

## Abstract

*Streptococcus pyogenes* (group A streptococcus, GAS) is a Gram-positive bacterial pathogen responsible for a wide variety of diseases. To date, GAS vaccine development has focused primarily on the M-protein. The M-protein is highly variable at the amino (N)-terminus (determining serotype) but is conserved at the carboxyl (C)-terminus. Previously a 29 amino acid peptide (named J14) from the conserved region of the M-protein was identified as a potential vaccine candidate. J14 was capable of eliciting protective antibodies that recognized many GAS serotypes when co-administered with immuno-stimulants. This minimal epitope however showed no immunogenicity when administered alone. In an attempt overcome this immunological non-responsiveness, we developed a self-adjuvanting vaccine candidate composed of three components: the B-cell epitope (J14), a universal helper T-cell epitope (P25) and a lipid moiety consisting of lipoamino acids (Laas) which target Toll-like receptor 2 (TLR2). Immunological evaluation in B10.BR (H-2k) mice demonstrated that the epitope attachment to the point of lipid moiety, and the length of the Laa alkyl chain have a profound effect on vaccine immunogenicity after intranasal administration. It was demonstrated that a vaccine featuring C-terminal lipid moiety containing alkyl chains of 16 carbons, with P25 located at the N-terminus, and J14 attached to the side chain of a central lysine residue was capable of inducing optimal antibody response. These findings have considerable relevance to the development of a broad spectrum J14-based GAS vaccine and in particular provided a rational basis for peptide vaccine design based on this self-adjuvanting lipopeptide technology.

## Introduction

Group A streptococcus (*Streptococcus pyogenes*, or GAS) is a Gram-positive bacteria that causes various diseases ranging from the relatively benign pharyngitis (‘strep throat’) to more invasive *necrotizing fasciitis* [1]. The post-infectious complications arising from GAS such as rheumatic fever (RF) and rheumatic heart disease (RHD) are responsible for the greatest health burden by causing the majority of morbidity and mortality (estimated at 12 million cases annually with 380,000 fatalities) [2]–[3]. Inadequate or delayed treatment of GAS infections can result in the development of these diseases and highlights the need for a protective GAS vaccine [4].

Development of a vaccine against GAS infections focused on the M-protein, an abundant cell surface protein. The M-protein is expressed by all GAS and is a major virulence factor [4]. Protective immunity against GAS infections has been associated with antibodies directed against the N-terminal or C-terminal regions of the M-protein [4], [5]. Protected individuals produce systemic IgG antibodies reactive to M-protein, enhancing phagocytosis and killing of the bacterium [4], [6]. The hypervariability of the N-terminal region made it difficult to develop a successful global vaccine for all serotypes of GAS. However an N-terminal based approach could be well suited for vaccines targeting specific GAS serotypes in particular geographical locations [7]–[9]. A vaccine strategy based on the conserved C-terminal region of the M protein can overcome these restrictions [4], [6], [10]–[13].

We have previously identified a peptide named J14 (KQAEDKVK***ASREAKKQVEKALE***QLEDKVK) containing 14 amino acids from the conserved GAS M-protein C region (shown in bold) enclosed within non-streptococal peptide sequences, designed to retain the native alpha-helical structure [14], [4]. This propensity to form a helix was critical for functionality as the immunodominant epitope displayed within J14 is alpha-helical in conformation [15].

**Figure 1:**
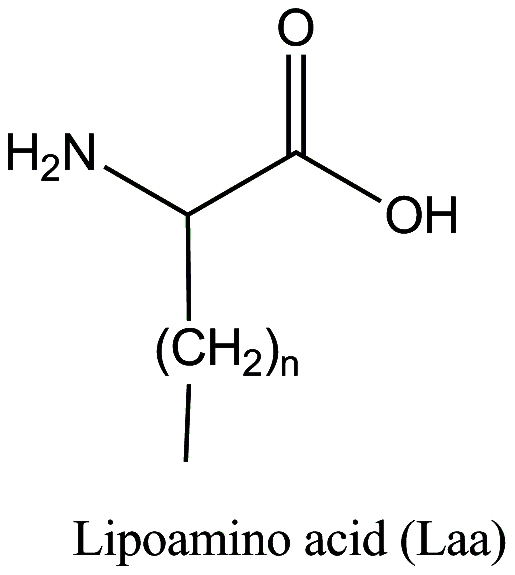
The structure of lipoamino acid (Laa). Lipoamino acids are described according to the total number of carbon atoms in the molecule. C12 Laa; n = 9. C14 Laa; n = 11. C16 Laa; n = 13.

Short synthetic peptides such as J14 are not sufficiently immunogenic on their own necessitating the use of adjuvant [4]. Bacterial lipoproteins/lipopeptides have been shown to enhance immune responses to otherwise weak immunogens comparable to the classical oil in water emulsion generated using Freund's complete adjuvant (CFA) [16]. Chemical conjugation of synthetic (including lipoamino acids or Laa, Figure 1) or bacterial derived lipids also demonstrated a high degree of immunogenicity with few or no side effects [16]–[20].

The potent adjuvant activity of these lipopeptides was due to the recognition of the lipid tail by the pathogen recognition receptor Toll-like receptor-2 (TLR2) [18], [19].

**Figure 2:**
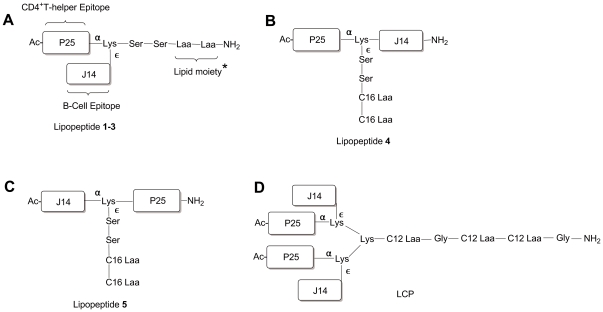
Structure of the lipopeptide vaccine candidates grouped according to the orientation of epitopes and Laa in the current study. * Lipopeptide 1 (C16 Laa), Lipopeptide 2 (C14 Laa), Lipopeptide 3 (C12 Laa).

To elicit antibodies against J14 without use of toxic adjuvants, we developed fully synthetic self-adjuvanting vaccine constructs (Figure 2a), composed of (i) a universal helper T-cell epitope (P25), (ii) the target GAS B-cell epitope (J14), and (iii) a lipid moiety based on Laa targeting TLR2. Since we are using racemic Laa, diasteromeric lipopeptides were utilized in the current study. The T-helper cell epitope (P25) was used to generate the proliferation of T cells to deliver help for antibody responses.

Varying the position of epitopes (J14 and P25) and the lipid moiety significantly affected the systemic J14-specific IgG response following intranasal administration (Lipopeptides **1**-**5,** Figure 2a–c). In order to optimize the adjuvanting activity of the lipid moiety, the effect of alterations in the length of Laa was also investigated (Figure 2a). The J14 and P25 peptides were also incorporated into a lipid core peptide (LCP) system (Figure 2d).

## Materials and Methods

### I. Lipopeptide synthesis

All constructs (Figure 2a-d) were synthesized using standard BOC chemistry [21]. Synthesis and physiochemical characterization of lipopeptides and LCP was previously described in detail elsewhere [21]. Briefly, for synthesis of lipopeptide **1**, two cycles of racemic Boc-protected C16 Laa was coupled to the resin, followed by two cycles of Boc-Ser(Bzl)-OH, and one Boc-Lys(Fmoc)-OH residue. Following Boc-deprotection of the lysine α-amine, the P25 peptide (sequence, KLIPNASLIENCTKAEL) was synthesized followed by N-terminal amine acetylation. The central lysine epsilon (ɛ) -Fmoc group was then removed, followed by the synthesis of the J14 sequence. For synthesis of diasteromeric lipopeptide **2-3**, two cycles of the appropriate Boc-protected Laa were coupled to the resin followed by the same synthesis procedure as lipopeptide **1** for attaching P25 and J14. For lipopeptides **4-5**, P25 was synthesized either N or C-terminally to J14. In both cases, Boc-protected C16 Laa was attached to the ɛ-amino group of the intervening lysine residue placed between the 2 epitopes.

### II. Intranasal immunization

Four to six week old female B10.BR mice (Animal Resource Centre, Perth, Western Australia) were intranasally immunized with 60 µg of diastereomeric lipopeptides **1-5** and diastereomeric LCP dissolved in 30 µL (15 µL/nare) of phosphate-buffered saline (PBS), followed by similar booster doses on days 21 and 41 post-primary immunization. Mice to be immunized were anesthetized first with a mixture of xylazine and ketamine (1∶1∶10 mixture of xylazine:ketamine:H_2_O; Provet, Brisbane, Australia). The positive control received 30 µg of J14 conjugated to the carrier protein Diptheria toxoid (DT) emulsified in a total volume of 50 µL of CFA subcutaneously at the tail base (J14-DT/CFA). Negative controls were given 50 µL of either PBS alone or 30 µg of DT in a total volume of 50 µL of CFA subcutaneously at the tail base. Negative controls were boosted with 50 µL of PBS and the positive control was boosted with 30 µg of J14 in a total volume of 50 µL of PBS. All animal protocols used were approved by the Institute's ethics committee (Griffith University Research Ethics Review Board for Animal-Based Work, GU Ref No: BDD-06-10-AEC) in accordance with National Health and Medical Research Council (NHMRC) of Australia guidelines.

### III. Collection of serum

Serum was collected on days 20, 40 and 60 post-primary immunization to determine the level of J14-specific systemic antibodies. Blood was collected from mice via the tail artery and allowed to clot for at least 30min at 37°C. Serum was collected after centrifugation for 10 min at 1000 g, heat inactivated for 10 min at 56°C and stored at −20°C.

### IV. Determination of antibody titers by ELISA

An enzyme linked immunosorbent assay (ELISA) was used to measure J14-specific serum IgG titers as described elsewhere [21]. Tested lipopeptides were compared to the LCP analogue, PBS administered intranasally and to J14-DT/CFA and DT/CFA administered subcutaneously. The titer was described as the lowest dilution that gave an absorbance of >3 standard deviation (SD) above the mean absorbance of control wells (containing normal mouse serum immunized with PBS). Statistical significance (*p*<0.05) was determined using a one-way analysis of variance (ANOVA) with Tukey post hoc test.

For the IgG subtype determination, mice sera from each group was pooled together and ELISA was done for the four antibody isotypes in duplicates for each group. Horse radish peroxidase-conjugated sheep anti-mouse IgG1, IgG2a, IgG2b and IgG3 antibodies were used as the secondary antibodies.

### V. Cell culture

Human embryonic kidney (HEK293) cells were cultured at 37 °C in 5% CO_2_ in Dulbecco's modified Eagle's medium (DMEM) containing 10% heat-inactivated serum supreme (BioWhittaker, Maryland, United States of America), 2 mM L-glutamine (L-Gln), 1 mM sodium pyruvate, and 500 µg/ml G418 sulfate (Geneticin). Cells were maintained in BD Falcon™ 75 cm^2^ cell culture flask and passaged every 3–4 days. The HEK293 cells were a gift from Dr. Ashley Mansell, Monash Institute of Reproduction and Development, Victoria, Australia.

### VI. HEK293 cell transfection and stimulation

HEK293 cells stably expressing TLR2 was assayed for their responsiveness to lipopeptides **1-3** and **5**. HEK293 cells were transfected with 1 µg of pNFκB-Luc Cis-Reporter plasmid per 1×10^6^ cells. pNFκB-Luc encodes firefly luciferase as a reporter gene for activation of the transcription factor NFκB. Consequently, increased luciferase activity in transfected HEK293 cells was directly proportional to the TLR2 mediated NFκB activation. Medium was changed to OPTI-MEMI medium (Invitrogen, California, United States of America) just before transfection and the transfection mixture was made by diluting 150 µl of 1,2-Dioleoyloxy-3-trimethylammoniumpropane chloride (DOTAP chloride) and L-alpha dioleoyl phosphatidyl ethanolamine (DOPE) in a 1∶1 ratio in 150 µl of OPTI-MEMI medium. 6 µg of plasmid DNA in 134.4 µl of OPTI-MEMI was then added to the transfection mixture. After a 10-min incubation at room temperature, the DNA-transfection mixture was diluted with 2.55 ml of OPTI-MEMI medium to afford addition of DNA-Lipofectamine mixture at 500 µl per well. Whilst only 5 wells were transfected, excess DNA was used to account for possible loss of DNA or pipetting errors.

After incubation for 24 h at 37°C in 5% CO_2_, the cells were seeded into 24-well plate (1.5×10^5^ cells/well) and the medium was refreshed to DMEM (along with 10% heat-inactivated serum supreme, 2 mM L-Gln, 1 mM sodium pyruvate) and incubated overnight. Post transfection (44 hr), triplicate wells were stimulated with diastereomeric lipopeptides **1-3** and **5** at 200 nM, 1.4 µM and 10 µM to deduce dose response. As controls, triplicate wells were stimulated with the TLR2 ligand dipalmitoyl-*S*-glyceryl cysteine (Pam_2_Cys, gift from Dr. Michael Batzloff, Queensland Institute of Medical Research, Queensland, Australia) at a concentration of 1.4 µM and medium alone (DMEM, 10% heat-inactivated serum supreme, 2 mM L-Gln, 1 mM sodium pyruvate). After 4 hr, cells were lysed and assayed for luciferase activity. Each experiment was repeated twice.

### VII. Luciferase reporter assay for NFκB activity

HEK293 cells were assayed for luciferase activity using the Dual-Glo luciferase assay kit (Promega, Wisconsin, United States of America). Cells were washed twice with 100 µl of PBS and lysed in 100 µl of passive lysis buffer (provided with the kit). The cell lysate was then centrifuged at 12470 *g* for 30 seconds at room temperature, and 10 µl of the supernatant was mixed with 50 µl of firefly luciferase substrate (luciferine) in a luminometer plate. The light illuminated was calculated using an illuminometer (Turner Designs, California, United States of America). The luciferase activity of each sample was normalized to the concentration of solubilized protein via the Bio-Rad Protein Assay (Bio-Rad, California, United States of America). After addition of the protein assay dye to 5 µl of lysate supernatant in 200 µl of Milli-Q water, the differential color change was measured at an absorbance of 595 nm with a Bio-Rad Benchmark Microplate Reader. Experimental data was shown as the relative increases over those cells treated with medium only. Data are shown as means ± SD of three cultures run in a given experiment. Variation between groups were analyzed using the one-tailed Student's t test and were regarded statistically significant if the *p* value was < 0.05.

## Results

### 1. Antibody response to lipopeptides

**Figure 3:**
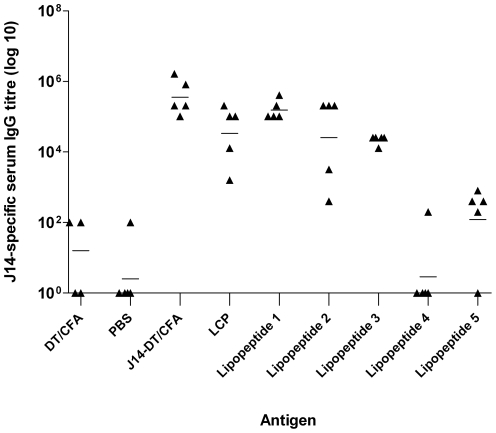
J14-specific serum IgG titers (log10) at the final bleed (day 60) after primary immunization for each individual mouse. Mean J14-specific IgG antibody titers are represented as a bar. Statistical analysis was performed using a one-way ANOVA followed by the Tukey post hoc test (ns, *p*>0.05; *, *p*<0.05; **, *p*<0.01; ***, *p*<0.001).

Following intranasal immunization, cohorts of mice administered lipopeptide **1** were shown to induce the highest J14-specific systemic IgG titers (Figure 3). These titers were significantly higher than the mice administered DT/CFA and PBS (Figure 3; lipopeptide **1** vs DT/CFA and PBS, *p*<0.001). The higher J14-specific IgG titers induced by lipopeptide **1** in comparison to the LCP system and analogues of **1** with shorter Laa alkyl chain length (Lipopeptides **2**, **3**) was not statistically significant (Figure 3; lipopeptide **1** vs LCP and lipopeptides **2**-**3**, *p*>0.05). Lipopeptide **1** induced comparable IgG titers to mice immunized with J14-DT/CFA (Figure 3; J14-DT/CFA vs lipopeptide **1**, *p*>0.05). Taken together, these data suggested that C16 Laa as in lipopeptide **1** was optimal for immunogenicity.

To investigate the effect of varying epitope and lipid orientation on J14-specific IgG titers, lipopeptides with the same C16 Laa but different epitope/lipid positioning were selected for comparison (Lipopeptides **1**, **4** and **5**). The point of lipid attachment significantly affected the J14-specific IgG antibody response (Figure 3; lipopeptide **1** vs **4-5**, *p*<0.001). Lipopeptide **1** (C16 Laa attached to the C-terminus) resulted in significantly higher antibody titers than lipopeptides **4** and **5** (where the lipid moiety was attached to the side chain ɛ-amine of the central lysine residue).

Lipopeptide **4** in comparison to **5** only differs in the orientation of the P25 and J14 epitope. The higher antibody titers observed for lipopeptide **5** in comparison to **4** was not statistically significant (Figure 3; lipopeptide **5** vs **4**, *p*>0.05)

**Figure 4:**
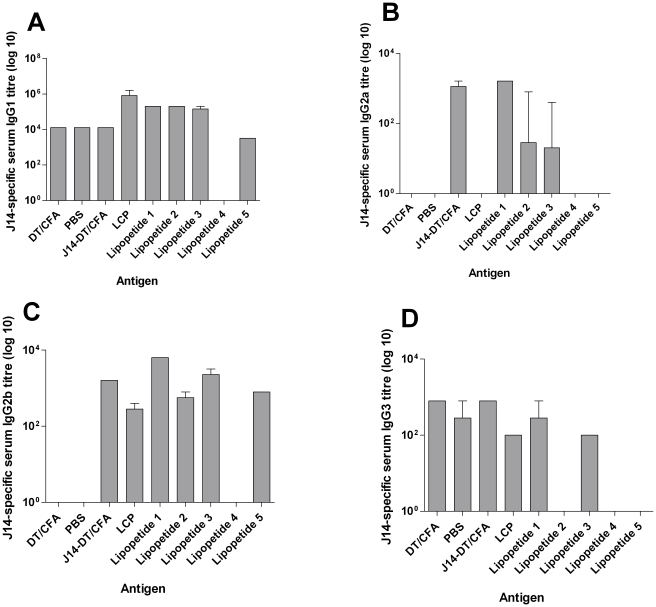
Isotypes of J14-specific serum IgG titers (log10) present in the final bleed (day 60) after primary immunization. Bars represent the titer for pooled sera obtained from each group. Error bars represent standard error of mean.

To further define the antibody responses, J14-specific IgG isotypes were analyzed. The most common IgG isotype observed for lipopeptides **1-3**, **5** and the LCP system was IgG1 (Figure 4a). Lipopeptide **1** elicited significantly higher IgG1 titers than mice administered lipopeptide **5** and J14+CFA/DT (Figure 4a; lipopeptide **1** vs lipopeptide **5** or J14-CFA/DT, *p*<0.001). Whilst the LCP construct elicited the highest IgG1 titre (Figure 4a), it should be noted that lipopeptide **1** was more immunogenic in view of the total IgG titre (Figure 3).

Following subcutaneous immunization with the positive control group J14-CFA/DT, predominantly IgG1 response was observed. Lower levels of IgG2a and IgG2b and antibodies were also elicited by J14-CFA/DT (Figure 4a–c).

Lipopeptide **1** in comparison to lipopeptides **2-5** and J14+CFA/DT elicited higher but not statistically different levels of IgG2a antibodies (Figure 4b; lipopeptide **1** vs lipopeptides **2**-**5** and J14-CFA/DT, *p*>0.05).

Lipopeptide **1** induced significantly higher IgG2b titers in comparison to mice administered lipopeptides **2**, **4**-**5** and the LCP analogue (Figure 4c; lipopeptide **1** vs lipopeptides **2**, **4**-**5** and LCP, *p*<0.001). Lipopeptide **1** induced IgG2b titers were also comparable to cohorts of mice administered J14-CFA/DT (Figure 4c; lipopeptide **1** vs J14-CFA/DT, *p*>0.05).

The few titers of IgG3 observed for lipopeptide **1** was comparable to levels of IgG3 titers induced by J14-DT/CFA or LCP (Figure 4d; lipopeptide **1** vs J14-DT/CFA and LCP, *p*>0.05).

Overall the IgG isotype data suggested that the trend for the lipopeptides was to induce preferential switching to IgG1 followed by IgG2b and IgG2a antibodies (T helper cell type 2 (T_H_2) biased response).

### 2. Lipopeptides with C16 Laa capable of targeting TLR2

**Figure 5:**
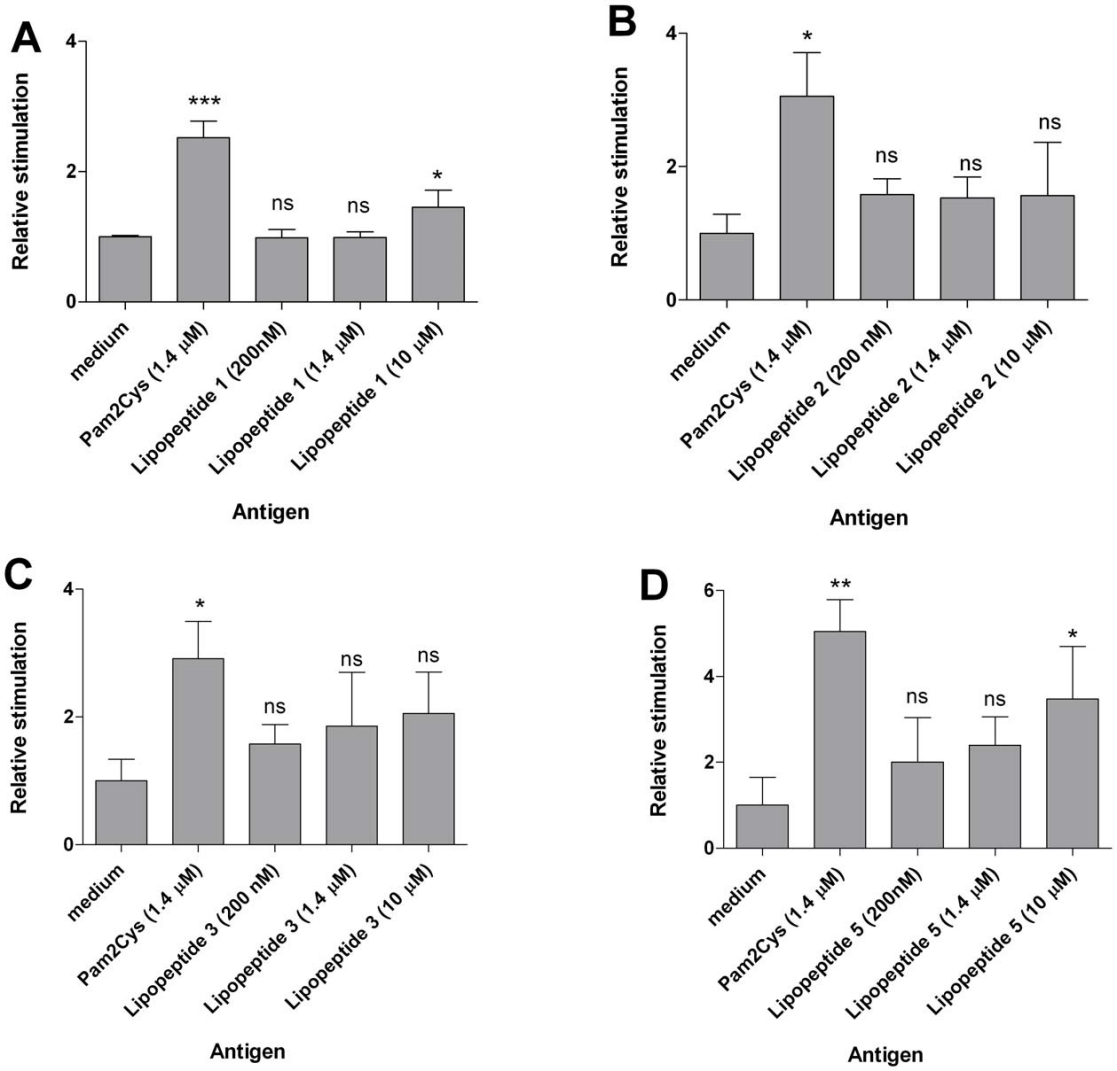
The ability of the lipopeptides to signal through TLR2 was tested by using HEK293 cells stably expressing TLR2 transfected with an NFκB-luciferase reporter gene. The several fold increase in NFκB levels in treated cells are shown relative to that of the TLR2-expressing cells treated with media, which was set to a value of 1. Bars are means ± SD of indicated number of experiments (ns, *p*>0.05; *, *p*<0.05; ** *p*<0.01; ***, *p*<0.001).

Two lipoamino acids provided our lipid moiety with two long alkyl chains, giving the lipid moiety structural similarity to the known TLR2 agonist Pam2Cys. Lipopeptide **1** with two copies of C16 Laa induced significant TLR2 stimulation at 10 µM concentration in comparison to cells treated with medium (Figure 5a). Lipopeptides **2-3**, which contained shorter alkyl chains in comparison to lipopeptide **1**, failed to induce significant TLR2 stimulation (Figure 5b, c). Lipopeptide **5** containing the same alkyl chain length as lipopeptide **1** but differing in position of the lipid moiety also induced significant TLR2 stimulation at 10 µM (Figure 5d).

## Discussion

Signaling through TLRs had been shown to result in robust and wide-ranging immune responses against peptide epitopes [18]. Our results suggested an alkyl chain length of 16 carbons for the Laa is optimal for stimulation of TLR2. The two ester bound fatty acids in Pam_2_Cys also contain chain length of 16 carbons which was said to be optimal for immunogenicity and activation of TLR2 [22], [23]. Lipopeptide **1** featured Laa with an alkyl chain of 16 carbons which may explain its ability to produce noticeable higher IgG titers in comparison to lipopeptides with shorter alkyl chain (Lipopeptides **2-3**).

Protection against GAS infection following vaccination is acquired by elicitation of high level of anti-GAS specific antibodies [24]. Lipopeptide **1** was shown to elicit comparable level of J14-specific IgG titers to mice administered J14 conjugated to DT and co-administered with CFA (a gold standard for adjuvant strength). Therefore immunization with lipopeptide **1** induced high level of anti-GAS specific antibodies which could confer protection against GAS infection. The selective induction of T_H_1 and T_H_2 cells can also mediate the protective immunity against certain diseases [25]. A T_H_2 response can provide protection against extracellular infection [26]. Therefore the T_H_2 biased response observed for our lipopeptides could be ideal for a protective immune response against GAS infection.

In the current study it was observed that positioning of the lipid moiety and peptide epitopes had a significant effect on immunogenicity. Our observation was that P25 at the N-terminus with J14 on the side-chain of the central lysine and a C-terminal lipid moiety with C16 Laa and was optimal for immunogenicity (lipopeptide **1**). The finding that incorporation of the lipid moiety and epitopes into different position influencing immunogenicity is in line with previous structure-activity studies [20]–[21]. Never the less the structure-activity relationship in the current study is important for designing successful peptide vaccines administered intranasally and provides another strategy for lipopeptide vaccine candidates based on Laa.

As the self-adjuvanting lipopeptides described in this study eliminated the use of any additional adjuvant and did not require conjugation to a carrier protein like DT, it can also overcome problems associated with peptide vaccines, including detrimental side effects of using more potent adjuvants and epitope suppression due to presence of antibodies to carrier proteins such as DT [21].

Paritcular features of lipopeptide **1** are also advantageous over the LCP system and other TLR2 targeting lipopeptides. Lipopeptide **1** contained one copy of each peptide epitopes and contains a lipid moiety that can be coupled to other amino acids without modifying standard peptide synthesis procedures (21). Thus it would be less expensive and easier to manufacture. The ability to synthesize lipopeptides in a highly pure, well-characterized state quickly and cheaply makes them desirable for use in human [26]. A shift from standard parenteral administration to the needle-free, non-invasive intranasal administration conferred by lipopeptides is also advantageous from a logistical, economic, and safety viewpoint [19], [22], [27].

The current paper described a rational approach to lipopeptide vaccine design by optimizing the alkyl chain length of the lipid moiety and position of the vaccine components for immunogenicity following intranasal administration. Immunological assessment of the lipopeptides identified a potential GAS vaccine candidate with the capacity to elicit high titers of systemic IgG which overcomes problems currently associated with the development of a vaccine against GAS. Overall, this work provided a rational basis for development of immunogenic lipopeptide vaccines for the prevention or treatment of many diseases and is of particular relevance to peptide based vaccines.
